# An inductive knowledge graph embedding via combination of subgraph and type information

**DOI:** 10.1038/s41598-023-48616-1

**Published:** 2023-12-01

**Authors:** Hongbo Liu, Yue Chen, Peng He, Chao Zhang, Hao Wu, Jiange Zhang

**Affiliations:** 1 Information Engineering University, Zhengzhou, 450001 China; 2 Zhengzhou University of Technology, Zhengzhou, 450004 China

**Keywords:** Computer science, Information technology, Scientific data

## Abstract

Conventional knowledge graph representation learn the representation of entities and relations by projecting triples in the knowledge graph to a continuous vector space. The vector representation increases the precision of link prediction and the efficiency of downstream tasks. However, these methods cannot process previously unseen entities during the knowledge graph evolution. In other words, the model trained on the source knowledge graph cannot be applied to the target knowledge graph containing new unseen entities. Recently, a few subgraph-based link prediction models obtained the inductive ability, but they all neglect semantic information. In this work, we propose an inductive representation learning model TGraiL which considers not only the topological structure but also semantic information. First, distance in the subgraph is used to encode the node’s topological structure. Second, the projection matrix is used to encode the entity type information. Finally, both kinds of information are fused for training to acquire the ultimate vector representation of entities. The experimental results indicate that the model’s performance has been significantly improved compared to the existing baseline models, demonstrating the method’s effectiveness and superiority.

## Introduction

A Knowledge Graph (KG) organizes and stores knowledge in the real world through multi-relationship-directed graphs, where nodes represent names of people, things, places and other entities, and edges represent relations between entities^1,2^ such as belonging and other attributes. Knowledge graph can also be seen as a collection of facts composed of triples (*h, r, t*). For instance, in the triple (*Bill Gates, founder, Microsoft*), *Bill Gates* and *Microsoft* serve as the head and tail entity respectively, and *founder* serves as the semantic relation between them.

With the gradual evolution of artificial intelligence from perceptual intelligence to cognitive intelligence in recent years.KGs are playing an increasingly important role in many domains, such as information retrieval^3^, question-answering systems^4^ and recommendation systems^5^. KGs have incompleteness because of two aspects. On the one hand, due to the incompleteness of people’s cognition of the real world and the limitations of data extraction algorithms, existing large-scale knowledge graphs often fail to capture and define all entities and relations. On the other hand, the knowledge graphs are ever-evolving, with new entities emerging every moment. Shi and Weninger^6^ performed a statistical analysis of DBpedia from late 2015 to early 2016 and found that about 200 new entities emerged daily. The common knowledge graph completion methods are embedding-based methods, such as TransE^7^, Complex^8^, and Rotate^9^, which learn the embeddings of entities and relations from the known triples and then predict the missing entities or relations.

**Figure 1:**
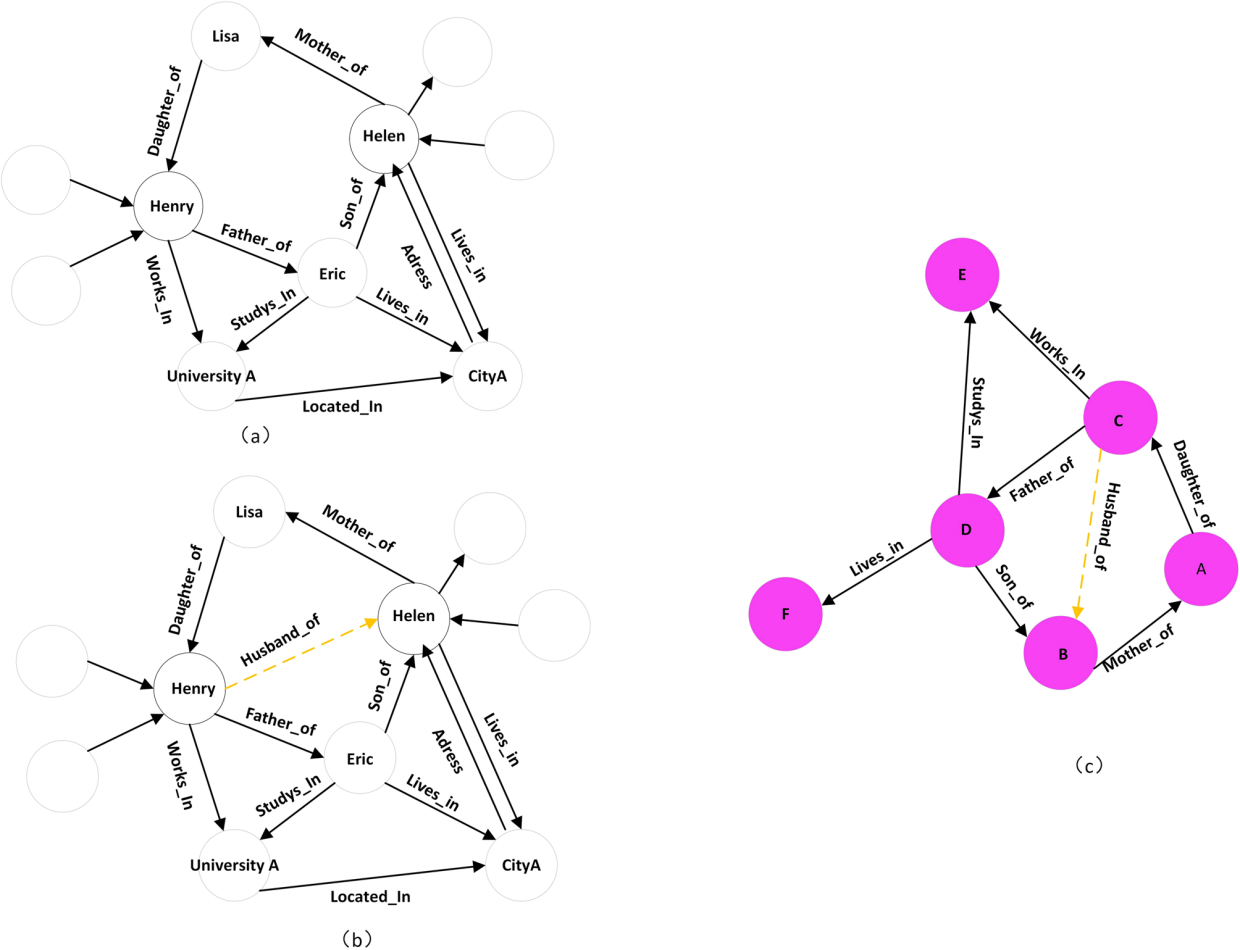
An example of relation prediction.

*Unseen entities* refer to the entities that are not included in the training set, but emerge in the testing set. In Fig. 1, the entities in Fig. 1a and b are the same, the entities in Fig. 1c are all unseen entities (different from those in Fig. 1a,b). Meanwhile, the relationships between the entities are the same in Fig. 1a–c. We learn the graph structure and the semantic information in Fig. 1a, and then apply the model to Fig. 1c to predict the relationship between the unseen entities. As shown in Fig. 1a, we can obtain the representations of entities *Henry* and *Helen* through training, and the representation contains the latent information that *Henry* is the husband of *Helen*. So the representations can be used to infer the missing relation between them in Fig. 1b. However, the entities in Fig. 1c are all unseen in Fig. 1a, we cannot get the representations of these entities through training, so the conventional embedding methods cannot predict the missing relation in Fig. 1c. The way to obtain the corresponding representation of novel entities is to retrain the whole knowledge graph containing unseen entities. However, retraining frequently is extremely space-consuming and time-consuming. So a solution avoiding costly retraining is desirable.

An approach that does not need retraining and can be used for unseen entities is to adopt rules, such as ${\exists }$ $\alpha$ ($\alpha$, Father of, $\beta$) $\wedge$ ($\beta$,Son of, $\gamma ) \rightarrow$ ($\alpha$, Husband of, $\gamma$). Rules can capture entity-independent semantic information and be directly applied to new entities^10^. However, most rule mining methods are implemented based on path traversal, and the number of rules is exponentially related to the scale of the knowledge graph. Meanwhile, rules are more dependent on graph structure other than semantics, so their expressiveness is lower than that of representation learning. Another research for unseen entity representation learning is to use external resources (such as entity description and category information). These approaches benefit inductive learning. But additional computation on massive resources is required, and this process is time-consuming and not always feasible.

Inspired by the generalization of Graph Neural Networks (GNN)^11,12^, some methods for inductive link prediction have emerged, such as GraiL^13^ and its related methods. The task of inductive link prediction in these methods is more challenging since it aims at predicting missing links between entities in knowledge graph, where the entities during training and inference can be different.These methods first extract subgraphs from the knowledge graph, then use the topological structure information among entities and relations in the subgraphs to predict missing relations and achieve good results. However, these methods only consider the topological structure and ignore the semantic information of entities in the subgraph.

The type of entity is one kind of semantic information. Most knowledge graphs possess entity-type information, and some types are constructed with hierarchical structures, in which different granularities of semantic concepts are considered sub-types in different layers. Xie et al.^14^ propose that the relation between two entities is constrained by the types of entities. For a triple (*Helen Mirren, graduated from, Harvard University*), its head entity should belong to the category person, and the tail entity should belong to the category educational institution. Motivated by the phenomenon, we propose to model the type information of entities and learn semantic information from entity-type, and integrate the structure and semantics to learn the inductive representation.

In this study, we propose a novel inductive representation method TGrail, which improves the generalization ability of representation learning by incorporating category information and structured information based on the subgraph.

The following is a list of this paper’s main contributions:

- Introducing TGrail, a novel integrated inductive knowledge graph embedding model. This model integrates the topological information from subgraph and the semantic information from entity-type, the combination of these two kinds of information enhances the generalization of knowledge graph embedding.
- Introducing an entity hierarchical type information coding method, which addresses the different roles of different hierarchical types by increasing the weight of abstract types and decreasing the weight of concrete types.
- Our model outperforms all baseline approaches by evaluating TGrail and several previously benchmark models on two datasets FB15K-237, WN18RR.

## Related work

### Transductive embedding models

Knowledge Graph Embedding(KGE) methods aim to learn the distributed representation of the entities and relations by projecting the elements in the knowledge graph to a continuous vector space. In other words, KGE can convert the symbolic representation of knowledge into a numerical representation while maintaining the knowledge graph’s internal structure and semantic information^15^. Such embeddings can improve the calculation efficiency of complex semantic associations between entities and relations, which is significant for constructing, reasoning, and applying knowledge base. Moreover, it has been extensively applied in tasks such as relation extraction, question-answering, and recommendation systems.

The typical knowledge graph embedding models based on facts alone are the translational distance and tensor decomposition models. TransE^7^, TransD^16^, TransR^17^ and other translational distance models evaluate the rationality of a triplet by modeling the relation into the translation operation between entities in vector space. Tensor decomposition models consider the knowledge graph a third-order tensor, where the head entities, tail entities, and relations index the mode-1, mode-2, and mode-3 vectors of the tensor, respectively, and the values of each tensor element are used to indicate whether the corresponding fact triples hold. Examples of such models include RESCAL^18^, DistMult^19^, ComplEx^8^.

In addition to relying on the triplet information alone, some models use additional information to improve the accuracy of the representation. Type information is one kind of additional information. Hierarchical-type information found in entities is frequently constructed artificially. It contains rich semantic information and can be regarded as more accurate prior knowledge. So it is important for the learning of representations in the knowledge graph.

Meanwhile, the type information is generalized rather than specific for a certain entity. Introducing hierarchical type into the relation prediction task can improve the accuracy of relation prediction, especially for some entities with fewer training samples. For instance, the relation *place of birth* generally connects two distinct types of entities. The categories of head and tail entities correspond to the personality and location type, respectively. Several models are proposed to add type information of entities to existing embedding models. For instance, the TKRL^14^ model adds explicit entity types to TransE. JOIE^20^ model represents the knowledge graph as an ontology view (i.e., type information) and instance view (i.e., entity information) and encodes these two views jointly. TaRP^21^ model encoded type information and instance-level information as prior probabilities and likelihoods of relations, respectively, and combined them with Bayes’rule.

The above transductive embedding models learn their vector representations by updating the initial random vectors of entities and relations. In contrast, the new entities of the target knowledge graph have no initial vectors or training process. They cannot be inferred by any other entities^3^. Therefore, the transductive embedding-based representation cannot solve the problem of new emerging entities’ representation in the knowledge graph.

### Inductive embedding models

Several kinds of inductive embedding models are proposed to solve the problem of new emerging entity representation, such as graph neural network-based, subgraph-based, description information-based and rule-based approaches.

#### Textual description information-based methods

Entity descriptions involve abundant semantic information, which can be utilized as auxiliary information to improve the accuracy of embedding learning. A few existing embedding-based models with such information integrated have shown success. Zhen W et al.^22^ proposed jointly embedding entities and words by aligning Wikipedia anchors and entity names into the same vector space. The DKRL model^23^ suggests using a convolutional neural network or continuous bag-of-words model to encode the textual information and then concatenating the text vector with the structure vector which is acquired from Trans model. Wang et al.^24^ proposed constructing a co-occurrence network combined with the entity and annotating in the corpus to achieve text-enhanced knowledge representation. Although these methods can achieve the representation of unseen entities out of the knowledge graph, they have some limitations. One is that the representations heavily rely on the presence of textual description information. The other is that two elements of the triple in which the new entity is located must both be in the knowledge graph. Therefore, these methods cannot be applied to the knowledge graph which cannot meet the above conditions.

#### Graph neural network-based methods

Graph neural network-based approaches acquire the representation of unseen nodes through aggregating neighbor nodes information, such as LAN^25^. However, these methods need the information of nodes around the unseen node and cannot be applied to an entire new graph composed of all unseen nodes.

#### Subgraph-based methods

The subgraph-based approaches, such as GraiL, TACT^26^, and CoMPILE^27^ , extract an enclosing subgraph surrounding the target relation firstly, then annotates the relative position of each entity in the subgraph, and design a score function using GNN for the annotated subgraph. the subgraph in our paper is composed of important nodes selected from the nodes around the target relation. There are several approaches for estimating the importance of nodes. The methods in GraiL, TACT and CoMPILE assume the distance from the target relation reflects the importance of the nodes. Other works such as PR and PPR take the probability of a node wandering randomly in the graph as the importance score. Recently a graph neural network-based method GENI is proposed. It applies an attentive GNN for predication-aware score aggregation to capture relations between the importance of the nodes.

CoMPILE emphasizes the directed nature of the edge in enclosing the subgraph and the message interactions between edges and entities. TACT addresses the semantic correlations between relations.These methods are all processed inside the enclosing subgraphs, neglecting the situations where the subgraphs are sparse and the relation prediction between the subgraphs. To solve these problems, SNRI^28^ proposes fully using the complete neighboring relations from the neighboring relational feature of the node and the neighboring relational path of the sparse subgraph. DEKG-ILP^29^ predicts the link between two subgraphs using the relation features based on contrast learning and the GNN-based subgraph features.

The training and testing set of these models has a disjoint set of entities, which means that these methods can learn the representation of unseen entities without restriction on seen entities. However, they only consider the topological structure of nodes in subgraphs, ignoring semantic information such as node types.

#### Rule-based methods

Rule-based methods learn a set of rules from the training data, and these rules are entity-independent, so they can be used for tasks that contain unseen entities, avoiding the trouble of retraining. According to the different strategies in the mining process, the rule learning methods are divided into path traversal-based methods, representation-based learning methods, and differentiable rule mining methods.

The RuleN^30^ is one of the path traversal-based methods. It first finds all triples (*a, r, b*) containing target relation *r*, searches all paths between *a* and *b* in the graph with a depth-first search strategy, and uses these paths as a body to form rules.

Tensorlog^31^, NeuralLP^32^, and DRUM^33^ are differentiable rule learners. The model Tensorlog establishes a connection between first-order rule inference and sparse matrix multiplication and compiles some specific logical reasoning tasks into a series of numerical matrice operations that can be differentiated. Based on Tensorlog, Neural LP propose a rule-learning framework that combines parameter and structure learning of first-order logic rules in an end-to-end differentiable model. Neural num-LP^34^ is an extension of the NeuralLP method, which adds numeric properties to the rule body based on NeuralLP. DRUM learns logical rules by establishing connections between the confidence scores of the rule and low-rank tensor approximation.

However, the rules have some limitations on expressing complex semantic correlations. Meanwhile, the number of rules is limited by the scale of the knowledge graph because of the consideration of search cost.

Compared to the aforementioned work, our work is mostly devoted to the problem of entity representation in entirely new knowledge graphs. We propose a knowledge graph inductive representation method incorporating subgraph structural features and entity-type semantic information.

## TGraiL: an inductive representation learning

### Problem definition

A knowledge graph is defined as G = (E,R,T), where E denotes the set of entities (containing head and tail entities), R is a set of relations between entities, and T is a set of all triples. In inductive knowledge Graph Embedding, we are given a source knowledge graph and a target knowledge graph, the source knowledge graph is defined as $\hbox {G}_s$={($\hbox {E}_s$,$\hbox {R}_s$,$\hbox {T}_s$)}, where $\hbox {E}_s$ is a set of entities (including head and tail entities), $\hbox {R}_s$ is a set of relations between entities, and $\hbox {T}_s$ denotes the set of all triple facts(*h,r,t*). The target knowledge graph $\hbox {G}_t$={($\hbox {E}_t$,$\hbox {R}_t$,$\hbox {T}_t$)},where $\hbox {E}_s$ $\cap$ $\hbox {E}_t$=$\emptyset$,$\hbox {R}_t$ $\in$ $\hbox {R}_s$.

Conventional knowledge graph representation methods first embed entities and relations into a low-dimensional continuous vector space. Then they define a corresponding scoring function to measure the rationality of triples, before obtaining the vector representations by maximizing scores of the known facts.

The goal of inductive knowledge graph representation learning is to learn representations of entities and relations in $\hbox {G}_s$, generalize them to target KG $\hbox {G}_t$, and use them to solve the inference problem of completely unseen entities in $\hbox {G}_t$. This paper evaluates the representations by triple classification and link prediction.

### Overall architecture of the model

**Figure 2:**
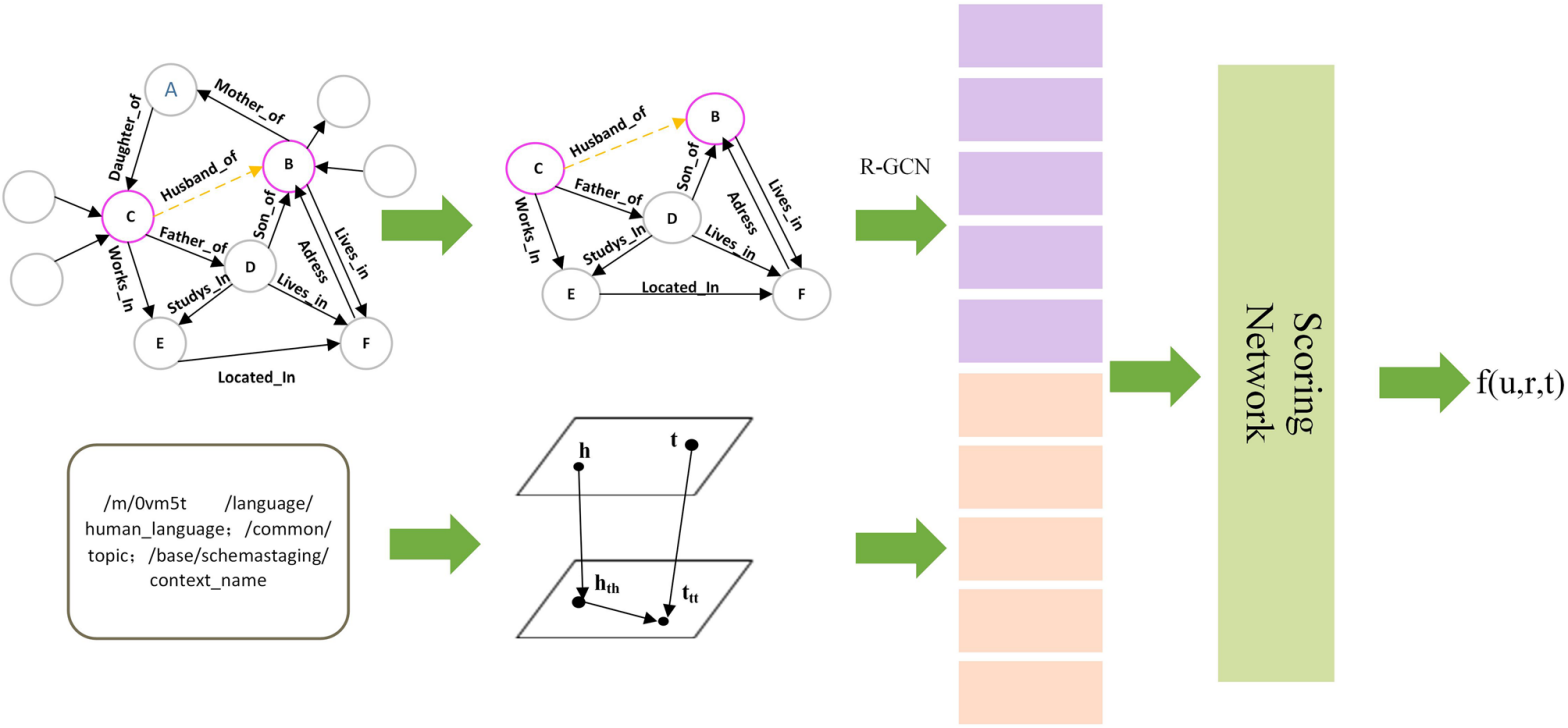
An overview of TGraiL.

The model TGrail is discussed in this section. Figure 2 depicts its architecture as a whole. The model consists of two parts, the type representation module and the topology representation module.

The type representation module encodes the hierarchical type of entities by constructing a projection matrix based on TKRL model. The topology representation module first extracts the directed subgraphs around the target relations. It uses the node topology in the subgraphs to obtain the subgraph representation based on the RGCN^35^ model training. A score function created by TGraiL integrates the two parts in a unified framework. The model is explained in detail below.

#### Structure representation

Subgraph Extraction

The relation *r* in the triple (*h,r,t*) is related not only to *h* and *t*, but also to the nodes and edges surrounding it. The quantity of nodes and edges around a particular relation *r* is huge in large-scale knowledge graphs. If all of them are considered, it is equivalent to aggregating all the node information in the whole knowledge graph, which may enhance the effect at the expense of impractical time and memory consumption for the majority of actual networks. The model SEAL^36^suggests that the high-order characteristics of the graph can be learned from the subgraph features within a small range around the target relation *r*. Therefore, this paper considers extracting the graph composed of nodes and edges within two hops around the relation *r* as enclosing subgraph, and subsequent works are based on the subgraph.

Existing WLNM^37^, SEAL, and GraiL models assume that the graph is undirected when extracting subgraphs, and all relations in the undirected graph are symmetric. However, not all relations in practice are symmetric. For instance, in triple (*Mike, parent, Lisa*), *Mike* is *Lisa’s* parent but *Lisa* is not *Mike’s* parent. The relation *parent* does not satisfy the symmetric. An incorrect result will be produced if the relation parent’s edge is treated as undirected. Accordingly, considering the nodes around the edge when extracting subgraphs is not enough, and their direction should also be considered. Similar to the concept of in- and out-degrees in directed graphs, the present study introduces incoming and outgoing nodes to define directed enclosing subgraphs.

##### Definition 1

For a node *v* in a directed graph, if there is an edge of length one from node *u* to *v*, then *u* is called the first-order ingoing node of *v*, and *v* is the first-order outgoing node of *u*. Similarly, *u* is referred to as the second-order ingoing node of *v*, and *v* is referred to as the second-order outgoing node of *u* if there is an edge with length two between *u* and *v*.

##### Definition 2

For a given triple (*u, r, v*), let $\hbox {S}^{1}_{in}$(h) be the collection of all lst-order in-going nodes of node *v*, $\hbox {S}^{1}_{out}$(h) be the collection of all lst-order outgoing nodes of node *u*, then the directed closed subgraph regarding *h* and *t* $\hbox {S}_{close}$= $\hbox {S}_{in}$(h)$\cap$ $\hbox {S}_{{out}}$(t) .

Node Representation

Similar to GraiL, we adopt DRNL^36^ to initialize the node embedding, which depicts the topological position of each node in the subgraph. For a target relation *r*, the head *u* and tail entity *v* which was linked by the relation *r* were seen as the target nodes, The topological structure of any node *i* in the enclosing subgraph is represented by a tuple (*d (u, i), d (i, v)*), where *d(u,i)* denotes the shortest distance from node *i* to the head node *u*, and *d(i,v)* denotes the shortest distance from node *i* to the tail node *v*. Then the representation of node *i* can be obtained by vectorizing the tuple.

Subgraph Representation

In the enclosing subgraph $\hbox {S}_{{close}}$ , the subgraph node embedding is trained using the RGCN model, and the calculation formula is shown as follows:

1 $$\begin{aligned} h_i^{(l + 1)} = \sigma \left( {\sum \limits _{r \in R} {\sum \limits _{{v_j} \in Nv_i^{(r)}} {\frac{1}{{{c_{i,r}}}}W_r^{(l)}h_j^{(l)} + W_o^{(l)}h_i^{(l)}} } } \right) \end{aligned}$$

where R denotes the extracted subgraph’s set of all relations, and $Nv_i^{(r)}$ denotes the set of neighbors whose relation to the node is *r*. $\hbox {c}_{i,r}$ is used for normalization. $\hbox {W}_r$ is the weight parameter corresponding to the neighbors with relation *r*, $\hbox {w}_o$ is the weight parameter corresponding to the node itself, $\sigma$() is the activation function, and $\hbox {h}_i^{(l)}$ represents the embedding representation of the node $\hbox {h}_i$ at the lth level.

The pooling average of all nodes in the subgraph is used to represent the subgraph.

2 $$\begin{aligned} h_{_{{s_{close}}}}^L = \frac{1}{{\left| {{V_s}_{_{close}}} \right| }}{\sum \limits _{i \in {V_s}_{_{close}}} {{h_i}} ^L} \end{aligned}$$

where ${V_s}_{_{close}}$ is the set of nodes in the enclosing subgraph.

A target node vector, edge vector, and a subgraph representation vector concatenate structured representation.

3 $$\begin{aligned} h = {h_{{s_{close}}}} \oplus {h_u}^L \oplus {h_v}^L \oplus {e_{{r_t}}} \end{aligned}$$

where ${h_S}_{_{close}}$ denotes the subgraph, ${h_u}$ , ${h_v}$denotes the nodes, and ${e_{rt}}$ denotes the edges.

#### Type representation

The type of entity in the knowledge graph has a hierarchical structure, such as *actor/award winner/person*, which reflects varying levels of abstraction across different types. During entity embedding learning, subtypes at different hierarchy levels play different roles. In transductive learning, detail categories offer richer information for representation learning. However, as inductive representation learning emphasizes more on generalization, abstract category information provides a more significant aid to representation learning. This paper uses a hierarchy-based weighted encoding approach for type representation to capture and exploit the hierarchical structure. Suppose there is a hierarchical type t=$\{ {t^{(1)}} \rightarrow {t^{(2)}} \rightarrow \cdots \rightarrow {t^{(k - 1)}}\}$ , with subtypes of granularity from fine to coarse as ${t^{(1)}},{t^{(2)}}, \cdots {t^{(k - 1)}}$ , e.g., *actor/award winner/person*, the most specific type is *actor*. For a subtype ${t^{(i)}}$ in the hierarchical type *t* , we use a projection matrix ${M_t}^{(i)}$ to represent subtype ${t^{(i)}}$ . ${\beta _i}$ to represent the weight of the subtype. The projection matrice of hierarchical type t can be formalized as

4 $$\begin{aligned} {M_t} = \sum \limits _{i = 1}^m {{\beta _i}} {M_t}^{(i)} \end{aligned}$$

where *m* is the number of layers of the hierarchy, $M_t^{(i)}$ is the projection matrix of the i-th subtype ${t^{(i)}}$ , and ${\beta _i}$ is the weight corresponding to ${t^{(i)}}$ . It is assumed that the more precise the type, the lower the weight, So we introduce a novel approach which ${\beta _i}$ decreases in equal proportion to the increasing value of subtype ${t^{(i)}}$ :

5 $$\begin{aligned} \frac{{{\beta _i}}}{{{\beta _{i + 1}}}} = \frac{1}{\varepsilon } - 1 \end{aligned}$$

where $\sum \limits _{i = 1}^k {{\beta _i}} = 1$, $\varepsilon \in$ (0, 0.5). An entity has multiple different types, and the project matrix of the entity is obtained by weighted summation of several different types. For triple (*h*, *r*, *t*) , the project matrix ${M_{rh}}$ of the head entity about relation *r* is defined as:

6 $$\begin{aligned} {M_{rh}} = \frac{{\sum \limits _{i = 1}^n {{\alpha _i}{M_{{t_i}}}} }}{{\sum \limits _{i = 1}^n {{\alpha _i}} }},{\alpha _i} = \left\{ \begin{array}{l} 1,{t_i} \in {\mathrm{{T}}_{\mathrm{{rh}}}}\\ 0,{t_i} \notin {\mathrm{{T}}_{\mathrm{{rh}}}} \end{array} \right\} \ \end{aligned}$$

where the number of entity types is denoted by *n* , ${t_i}$ denotes the i-th type of entity *e* , ${M_{{t_i}}}$ is the projection matrix of ${t_i}$ , and ${\alpha _i}$ is the weight of ${t_i}$ . ${\alpha _i}$ can be obtained from the frequency of the entity belonging to ${c_i}$. ${T_{rh}}$ denotes the relation set of the head entity about relation *r* .

#### Framework of TGrail

In order to make full use of hierarchical type and structure information, we designed a score function to combine the two parts in a unified framework. The score function is

7 $$\begin{aligned} f(u,r,v) = [h\oplus {h_{{s_{close}}}}]{W_s} \end{aligned}$$

where *h* and ${h_{{s_{close}}}}$ are the embedding vectors of the hierarchical type module and structure representation module, respectively. In model training, the loss function in TransE is used as the objective optimization function of the training model. A binary classification task is performed on the given triple, and the goal is to maximize the distance between the closest positive and negative examples. The negative sample is constructed by randomly replacing the triple’s head (or tail) with a uniformly sampled entity.

8 $$\begin{aligned} \mathrm{{L = }}\sum \limits _{(u,r,v) \in G} {\max (0f(u',r,v') - f(u,r,v) + \gamma )} \end{aligned}$$

where $\gamma$ is the regularization parameter, *G* represents the set of all triples in the knowledge graph, (*u*, *r*, *v*) and $(u',r,v')$ denote the positive and negative triple respectively.

## Experiments

This section evaluates the model’s effectiveness in link prediction and triple classification. First,the experimental dataset configuration, evaluation metrics are described. Second, the experiment results of TGrail on several benchmark datasets are shown.

### DataSets

We perform experiments on two benchmark datasets WN18RR and FB15K-237.

The type information of the entities in FB15K-237 uses the category information provided in the literature^14^. The WN18RR dataset currently does not have dedicated category information, so the hypernym of the entities is used as the category information. The statistical data shows that 4596 entities do not have hypernym word information, accounting for about 11% of the total entities. In order to ensure the integrity of the dataset, the triplets involving these entities are preserved, and the missing entity categories are filled with the mode in the data processing. The inductive representation mainly focuses on the model’s performance on unseen entity representation. The more the number of unseen entities in the evaluation dataset, the more powerful the model’s evaluation ability. As a result, the experiment adopts the GraiL dataset division method, in which several nodes are randomly selected from KG as root nodes, and the graph composed of K-hop neighborhood nodes around the root node is used as the training graph. The test graph was generated similarly after removing the training graph from KG. Both datasets are divided into four parts, V1, V2, V3 and V4. The corresponding test sets are v1_ind, v2_ind, v3_ind and v4_ind. In order to verify the inductive characteristics of the model, there are no duplicate entities in the training and test sets, and the relations in the test graph are taken from the train graph. Detailed information about the training set and test set is shown in Table 1:

**Table 1:** Statistics of inductive benchmark datasets. #E , #R, and #TR are used to denote the number of entities, relations, and triples respectively.

		WN18RR			FB15K237		
		#R	#E	#TR	#R	#E	#TR
V1	Train	9	2746	6678	183	2000	5226
	Test	9	922	1991	146	1500	2404
V2	Train	10	6954	18,968	203	3000	12,085
	Test	10	2923	4863	176	2000	5092
V3	Train	11	12,078	32,150	218	4000	22,394
	Test	11	5084	7470	187	3000	9137
V4	Train	9	3861	9842	222	5000	33,916
	Test	9	7208	15,157	204	3500	14,554

### Evaluation metric

To keep consistent with the baseline model, we perform triple classification and link prediction tasks and adopt the AUC-PR and Hits@n as the evaluation metric.

Triple classification is a binary classification task to determine whether a given triple exists in a knowledge graph. This task scores each triple by a scoring function and sets a threshold for the score. If the score exceeds the threshold, the triple is considered correct. Otherwise, it is considered to be wrong. The classification task requires negative triplets, but there are no publicly released negative triplets in the current dataset, so we construct negative triplets by randomly replacing the triplet’s head entity or tail entity. We adopt AUC-PR as the evaluation metric for triple classification because this experiment emphasizes the model’s discriminative ability in positive samples.

Link prediction is to predict the third element in a triple based on the existing two elements. Entity prediction refers to predicting the missing entity *h* or *t* in the triplet, and relation prediction predict the missing relation *r* between two given entities. In this experiment, link prediction refers to relation prediction.

### Experimental setting

PyTorch^38^ is used to implement the model. Experiments are conducted on GTX 2080 Ti with a RAM capacity of 12 GB. The Adam^39^ optimizer was utilized with a batch size of 16 and a learning rate of 0.001. The number of training epochs is 20. The embedding vector dimension of entity, relation, and hierarchical type is set to 32, with default values for other parameters.

### Results

**Figure 3:**
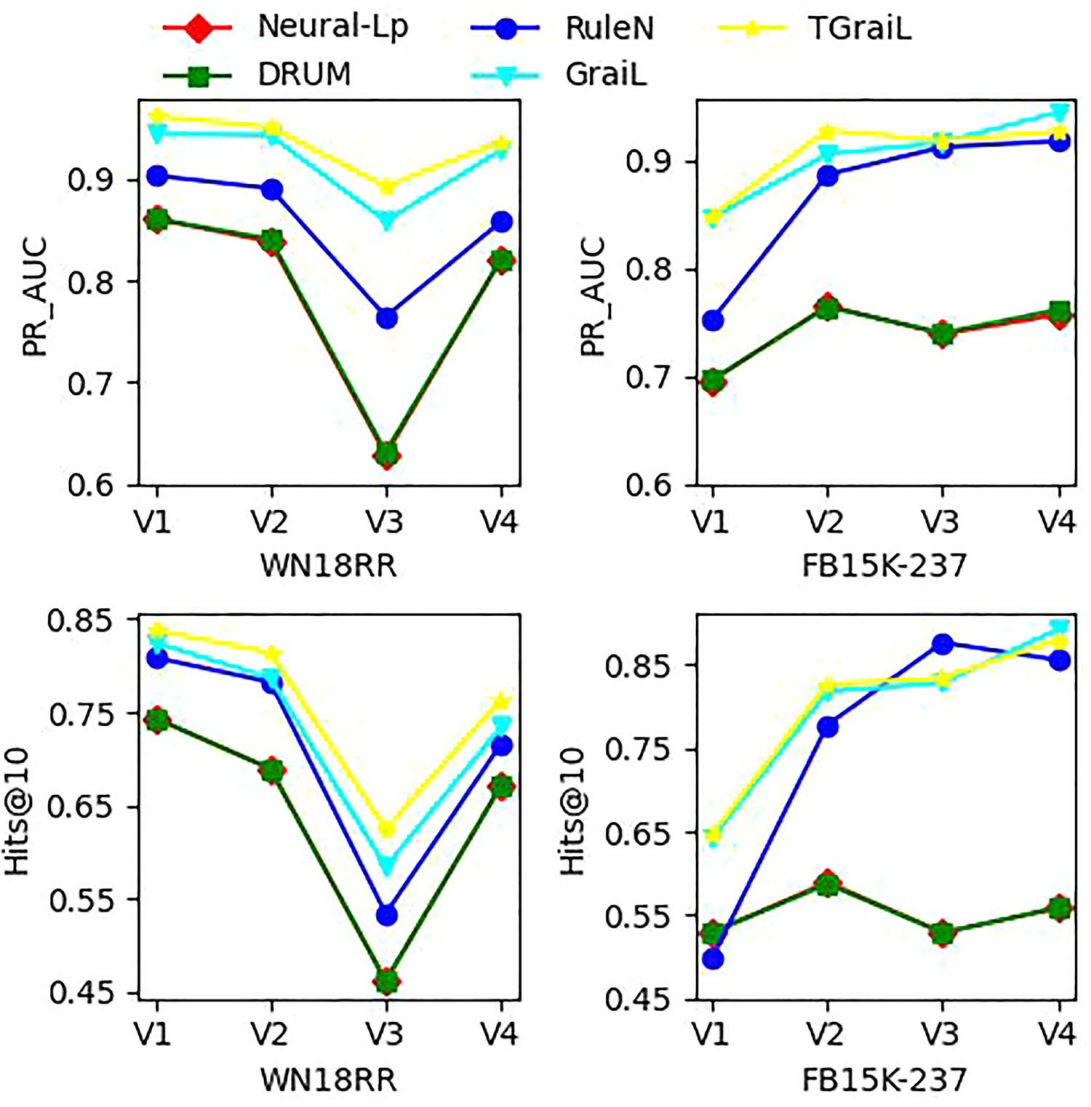
Performance variation of different models.

Tables 2 and 3 demonstrate the experimental results of AUC-PR and Hits@10 on v1, v2, v3 and v4 of WN18RR, FB15K-237. To better compare the performance of different models on Hits@10 and AUC-PR, we plotted the performance curve of all models on the two datasets, as shown in Fig 3.

Several phenomenons can be observed:

- Among the three rule-based methods, RuleN performs the best, two end-to-end differentiable methods NeuralLP and DRUM achieve similar performance.It indicates that the rule acquired by path-based mining has more inductive.
- For Hits@10 and PR-AUC, the results of GraiL are all better than NeuralLP on datasets WN18RR and FB15K-237. Compared to the two methods, we note that the sampling subgraph in RuleN is similar to the enclosing subgraph in GraiL. The difference is that the RuleN method performs rule mining based on the subgraph, while the GraiL method uses the graph neural network method for feature learning. The result demonstrates that GraiL learns not only the topological structure of each node’s neighborhood but also the distribution of node features in the neighborhood.
- TGrail outperforms all the inductive baselines for all metrics on WN18RR and FB15K-237. It suggests that the integration of type information is beneficial for inductive representation.

**Table 2:** Inductive results (PR_AUC).

	WN18RR				FB15K237			
	V1	V2	V3	V4	V1	V2	V3	V4
Neural-LP	86.02	83.78	62.90	82.06	69.64	76.55	73.95	75.74
DRUM	86.02	84.05	63.20	82.06	69.71	76.44	74.03	76.20
RuleN	90.26	89.01	76.46	85.75	75.24	88.70	91.24	91.79
GraiL	94.32	94.18	85.80	92.72	84.69	90.57	91.68	94.46
TGraiL	96.10	94.98	89.22	93.55	84.89	92.81	91.81	92.70

**Table 3:** Inductive results (Hits@10).

	WN18RR				FB15K237			
	V1	V2	V3	V4	V1	V2	V3	V4
Neural-LP	74.37	68.93	46.18	67.13	52.92	58.94	52.90	55.88
DRUM	74.37	68.93	46.18	67.13	52.92	58.73	52.90	55.88
RuleN	80.85	78.23	53.39	71.59	49.76	77.82	87.69	85.60
GraiL	82.45	78.68	58.43	73.41	64.15	81.80	82.83	89.29
TGraiL	83.78	81.41	62.48	76.35	64.63	82.69	83.46	87.96

### Comparison experiments

#### Comparison of different type encoding schemes

In order to analyze the influence of different entity type coding schemes for the inductive representation of the model, we introduce TGraiL-whe, in which the type coding adopts the WHE method in TKRL, and the hierarchical type weight changes proportionally according to the type inclusion range, the more specific the category, the greater the weight. Taking the example of the hierarchical type */music/artist*, it is observed that *artist* is the most specific entity and is assigned the largest weight. However, in TGraiL, greater emphasis is given to type generalization, so the subtype *artist* with more specific types is assigned smaller weights. As illustrated in Table 4, the effect of the TGraiL_whe in unseen entities is not as good as TGraiL, and it shows that different hierarchical weight calculation methods have different effects on the inductive representation of the model. Excessively detailed category information affects the accuracy of predictions.

#### Comparison of descriptive information and type information

To compare the different effects of description information and hierarchical type on inductive knowledge graph embedding, we design a baseline model GraiL_des, in which the entity description information is vectorized with BERT. Table 5 shows the results of the two models on the benchmark dataset FB15K-237. As illustrated in Table 5, Compared with GraiL, the performance of GraiL_des, which incorporates description information, drops. It indicates that the detailed description information limits the representation capability of the model on unseen entities, so the influence of type information is far better than description information on inductive knowledge graph representation.

**Table 4:** The Hits@10,PR_AUC results of the TGraiL_whe and TGraiL on FB15K-237.

	Hits@10				PR_AUC			
	V1	V2	V3	V4	V1	V2	V3	V4
TGraiL	64.63	82.69	83.46	87.96	84.89	92.81	91.81	92.70
TGraiL_whe	62.01	81.05	82.25	85.06	82.37	89.35	90.26	91.56

**Table 5:** The Hits@10,PR_AUC results of the GraiL_des and TGraiL on FB15K-237.

	Hits@10				PR_AUC			
	V1	V2	V3	V4	V1	V2	V3	V4
TGraiL	64.63	82.69	83.46	87.96	84.89	92.81	91.81	92.70
GraiL_des	61.46	77.69	76.48	82.75	80.25	84.32	89.68	92.20

## Conclusion

Transductive embedding models cannot generate representations for unseen entities that may emerge subsequently. Some inductive methods recently proposed realized inductive learning based on subgraph extraction and GNN. While these methods only account for structural characteristics related to nodes and their neighborhoods, overlooking the expressive semantics encapsulated within the hierarchy types of the nodes. To address the semantic information, we propose a novel inductive knowledge graph embedding method in this paper, which incorporates the subgraph structure information around the relation and integrates the category features. Experimental results indicate that the proposed TGrail performs better than several current state-of-the-art techniques on benchmark datasets.

However, the TgraiL method still has limitations. It only captures the structure and semantics of the subgraph, ignoring the topology structure in a global graph. In future work, we will explore how to extract the global features of the graph to enhance the generalization ability of inductive representation learning.

## Data Availability

The datasets used during the current study are available from the corresponding author on reasonable request.
